# ID 85 - Panorama da Formação e Produção Científica relacionada à Avaliação Econômica em Saúde no Brasil

**DOI:** 10.5327/2237-9622.2025.v34s1.85

**Published:** 2025-11-25

**Authors:** Ana Flávia de Morais Oliveira, Everton Nunes da Silva, Yara Andrade Marques, Jéssica Portal Maia, Ivan Zimmermann, Rodrigo Luiz Carregaro, Henry Maia Peixoto

**Keywords:** educação continuada, autoria, avaliação, econômica em saúde, Sistema Único de Saúde

## Abstract

**Introdução:**

A sustentabilidade do sistema público de saúde brasileiro é complexa e desafiadora, tendo em vista que se trata de uma vasta Rede de Atenção à Saúde (RAS) que atende mais de 70% da população, de modo gratuito e universal. Diante da crescente demanda por serviços de saúde, associada à limitação de recursos financeiros, diversos países têm fomentado a realização de estudos de avaliação econômica em Saúde (AES) como balizador para tomadores de decisão. Entretanto, há uma lacuna quanto ao panorama da AES no Brasil.

O objetivo do estudo foi caracterizar e detalhar a formação, e discriminar a produção científica em AES de pesquisadores brasileiros. Secundariamente, o estudo investigou a relação entre o quantitativo das produções e os investimentos financeiros em pesquisa em saúde nos últimos anos, no Brasil.

**Método:**

Foi realizado um estudo descritivo de base quantitativa, com informações obtidas por meio de dados cadastrados nos currículos de pesquisadores disponíveis na plataforma Lattes. A busca dos currículos deu-se por meio do uso de descritores, relacionados à AES. Foram incluídos no estudo os currículos dos pesquisadores brasileiros com formação nas áreas correlatas da saúde e da economia, com experiência profissional no campo da AES. A busca e a análise dos currículos ocorreram entre os meses de janeiro a junho de 2023.

**Resultados:**

Foram avaliados 2.534 currículos. Destes, 2.424 não atenderam aos critérios de inclusão, sendo incluídos 110 currículos. A maioria dos pesquisadores são graduados em medicina (n=41; 37,27%), farmácia (n=23; 20,91%) e enfermagem (n=15; 13,64%). A localidade de conclusão das graduações ocorreu em sua maioria na Região Sudeste (n=71;64,55%), o estado de São Paulo obteve o maior registro de graduação (n=29;26,36%), e a Região Norte representou o menor número de graduações no período (n=2;1,82%). Os pesquisadores concluíram o doutorado em diversas áreas. O maior número de registros entre os cursos foram: Ciências da Saúde (n=33; 30,00%) e Saúde Coletiva (n=17; 15,45%). Os cursos de doutorado aconteceram majoritariamente em instituições públicas no País (n=101; 91,82%). A atuação profissional dos pesquisadores está concentrada na Região Sudeste (n=72;65,45%). Os pesquisadores em análise apresentam o setor público como o seu principal local de atuação atual (n=87; 79,09%). Os pesquisadores em análise publicaram 1.147 artigos relacionados à AES. Verificamos que publicações envolvendo estudos do tipo custo-efetividade, utilidade, minimização e benefício foram as mais frequentes (n=472; 40,83%), seguido dos estudos de custo de doença, programa, carga da doença e outros (n=368; 32,08%).

**Conclusão:**

Os pesquisadores brasileiros em AES caracterizaram-se por obter como formação básica mais frequente a graduação em medicina, assim como o curso na modalidade stricto sensu (doutorado) em Saúde Coletiva. Em ambas as situações, a formação aconteceu, predominantemente, na Região Sudeste. A atuação dos profissionais também foi registrada com maior frequência na Região Sudeste. As informações reunidas no estudo têm o potencial de auxiliar na construção de políticas públicas que visem impulsionar a formação em AES e fomentar pesquisas nessa área. Isso, por sua vez, pode promover maior eficiência no sistema de saúde brasileiro, fortalecendo seus princípios doutrinários de universalidade, equidade e integralidade.

